# Paxlovid–tacrolimus drug–drug interaction caused severe diarrhea that induced combined diabetic ketoacidosis and a hyperglycemic hyperosmolar state in a kidney transplant patient: a case report

**DOI:** 10.1186/s13256-023-04135-1

**Published:** 2023-09-24

**Authors:** Wei Luo, Yu He, Mao Gang Wei, Guang Bing Lu, Qun Yi

**Affiliations:** 1 Department of Respiratory and Critical Care Medicine, The People’s Hospital of Leshan, Leshan, 614000 Sichuan People’s Republic of China; 2 Department of Critical Care Medicine, Meishan Traditional Chinese Medical Hospital, Meishan, 620010 Sichuan People’s Republic of China; 3 Department of Respiratory and Critical Care Medicine, West China Hospital of Sichuan University, Chengdu 610041 Sichuan, People’s Republic of China; 4 Department of Critical Care Medicine, Sichuan Cancer Hospital, Chengdu, 610041 Sichuan People’s Republic of China

**Keywords:** Coronavirus disease 2019, Transplant recipients, Immunosuppressant, Paxlovid, Tacrolimus, Diabetic ketoacidosis, Hyperosmolar hyperglycemic state, Case report

## Abstract

**Background:**

Transplant recipients are at high risk of coronavirus disease 2019, and a timely supply of antivirals should be prioritized for those patients. Complicated drug‒drug interactions limit the use of Paxlovid (nirmatrelvir/ritonavir) coadministered with tacrolimus. Here, we report a patient with a kidney transplant who received Paxlovid and reduced-dose tacrolimus at the same time and suffered a severe tacrolimus toxicity.

**Case presentation:**

We present a 56-year-old man of Han ethnicity with a kidney transplant who suffered from coronavirus disease 2019 twice. For the first infection, the immunosuppressants were substituted by dexamethasone when the patient used Paxlovid, and everything went well. For the second time, tacrolimus at a reduced dose concomitant with Paxlovid caused severe diarrhea, inducing combined diabetic ketoacidosis and a hyperglycemic hyperosmolar state.

**Conclusion:**

This case challenges the dose-adjustment strategy of managing drug‒drug interactions. We suggest that tacrolimus should be stopped when Paxlovid is applied and that corticosteroids could be a good substitution.

## Background

Paxlovid (nirmatrelvir/ritonavir) received emergency use authorization in 2021 to treat patients with mild to moderate coronavirus disease 2019 (COVID-19). Transplant recipients are definitely at high risk for COVID-19, and a timely supply of antivirals should be prioritized for those patients. Ritonavir, as a component of Paxlovid, has potential drug interactions with immunosuppressants, such as tacrolimus, based on the mechanism of the cytochrome P450 (CYP) 3A inhibitory effect [1]. Here, we report a patient with a kidney transplant who received Paxlovid and reduced-dose tacrolimus at the same time and suffered severe tacrolimus toxicity.

## Case presentation

**Fig. 1:**
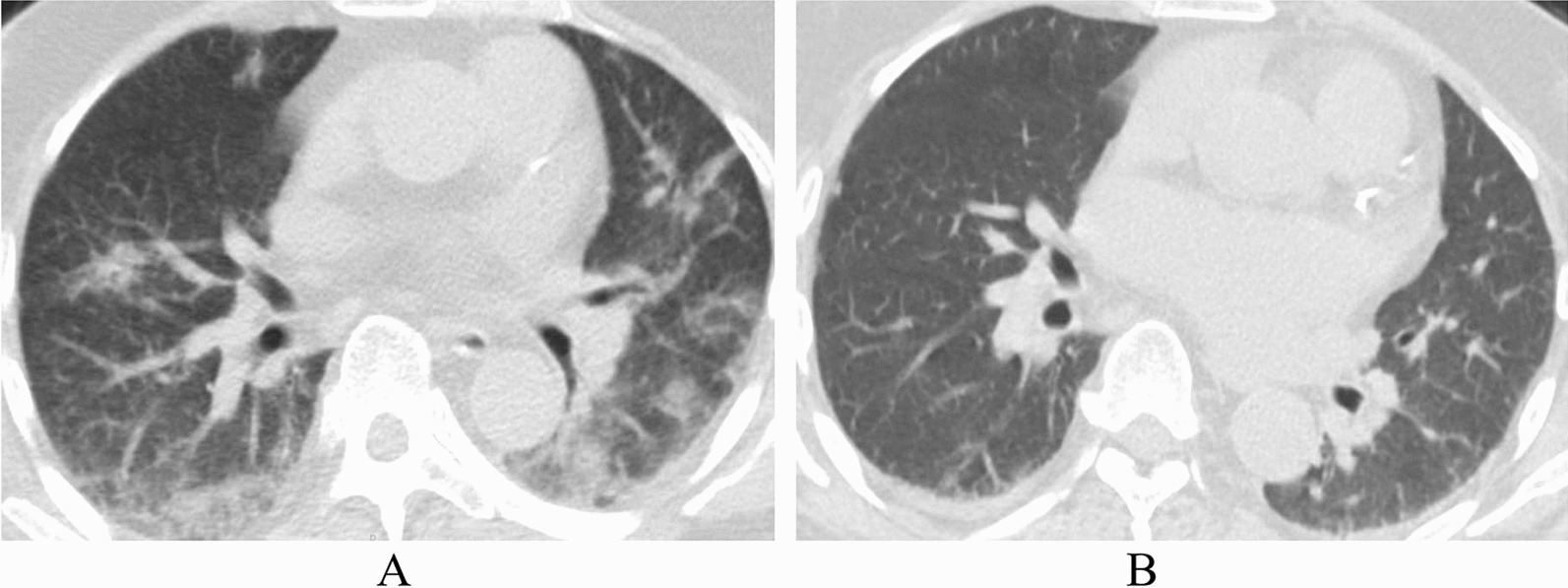
**A** The presence of ground-glass opacities in both lungs. **B** Normal thoracic computerized tomography scan

A 56-year-old male, who was ethnic Han, with a known history of type II diabetes mellitus and who received a kidney transplant 10 years previously was admitted to our department with the chief complaint of fever and dry cough. He denied shortness of breath or chest pain. His immunosuppression regimen included oral tacrolimus 2 mg twice daily (BID) (his tacrolimus blood concentration was 4.66 ng/mL one month prior) and oral mycophenolic acid 360 mg BID. Insulin was administered subcutaneously at regular intervals, and his blood glucose was controlled well. He experienced severe pneumonia caused by severe acute respiratory syndrome coronavirus 2 (SARS-CoV-2) 5 months prior (Fig. 1A). Paxlovid was administered, and dexamethasone was substituted as an immunosuppressant during that hospitalization. This patient was a businessman, and his social, environmental, family, and psychosocial history was unremarkable. He did not smoke or consume alcohol.

He was hemodynamically stable with an O_2_ saturation (SpO_2_) of 98% while breathing ambient air, and his physical exam was unremarkable. His laboratory values were significant for a serum creatinine (Scr) level of 186 μmol/L and a urea nitrogen (BUN) level of 15.85 mmol/L. The patient’s nasopharyngeal swab SARS-CoV-2 nucleotide test was positive. The thoracic computerized tomography (CT) scan was normal (Fig. 1B). Oral Paxlovid was administered as 300 mg of nirmatrelvir combined with 100 mg of ritonavir BID. His immunosuppressant drugs were adjusted to a quarter of the previous doses: oral tacrolimus 1 mg BID and oral mycophenolic acid 180 mg BID one day apart (Table 1). This patient responded well after 3 days of taking Paxlovid: his temperature returned to normal, and his cough was relieved. On the fourth day of taking Paxlovid, this patient suffered sudden severe diarrhea at night lasting nearly 8 hours, and he did not receive any medical intervention (he went home at night without permission). He was confused and short of breath when he came back. The physical exam revealed dehydration, a drowsy state, tachypnea (35 breaths per minute), and tachycardia (110 beats per minute). His blood pressure was 108/60 mmHg with an SpO_2_ of 95%. There were scattered crackles in the lung bases.

**Table 1:**
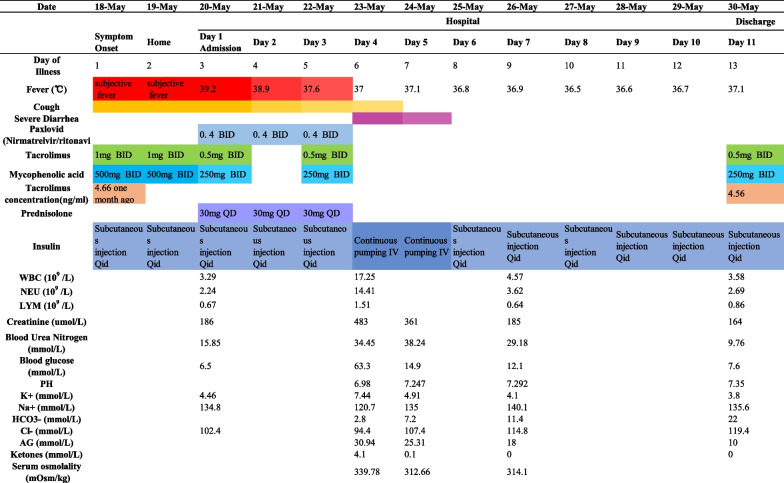
Symptoms and maximum body temperatures according to day of illness, day of hospitalization, laboratory tests, and drug use

**Fig. 2:**
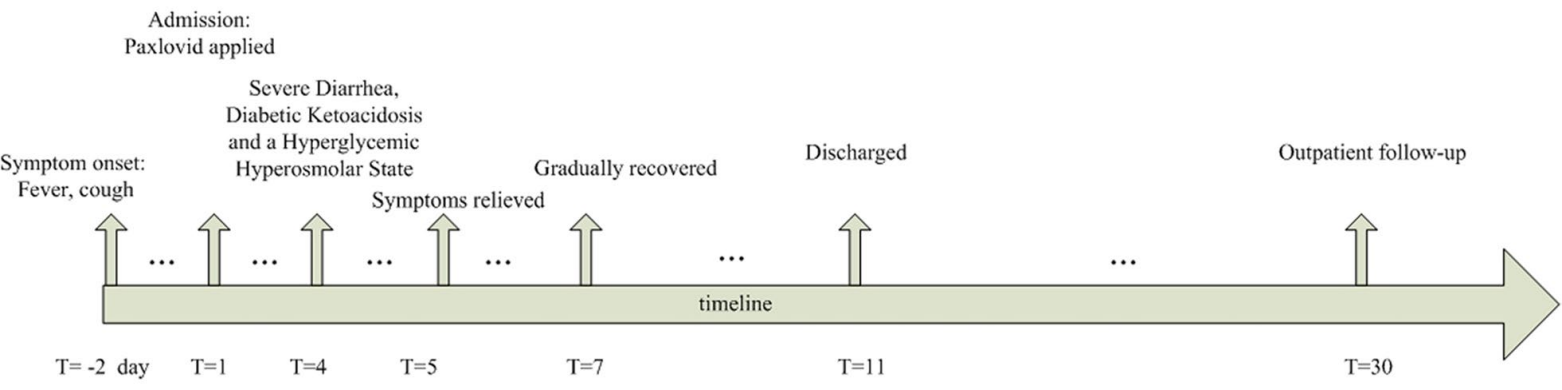
Timeline demonstrating important dates for the patient in hospital and on outpatient follow-up

Laboratory tests demonstrated that glucose was 63.3 mmol/L and that blood ketones had increased to 4.1 mmol/L. Blood gas analysis showed metabolic acidosis with a pH of 6.98 and bicarbonate of 2.8 mmol/L. The patient developed acute kidney injury with a serum creatinine (sCr) of 483 µmol/L and a BUN of 34.45 mmol/L. The calculated serum osmolality was elevated at 339.78 mOsm/kg. A broad work-up for diarrhea was carried out, including routine stool tests and culture, and all these tests were negative. Combined diabetic ketoacidosis and a hyperglycemic hyperosmolar state (HHS) were confirmed. Paxlovid and immunosuppressants were withheld. Loperamide 4 mg orally four times daily (QID) and montmorillonite powder 3 g orally three times daily (TID) were administered. Restoration of intravascular volume and correction of electrolyte abnormalities, acidosis and hyperglycemia were carried out. At 24 hours, the diarrhea stopped, and the blood glucose was approximately 15 mmol/L. Three days later, homeostasis was reestablished with almost normal pH, electrolytes, blood glucose, and blood ketones. Over the next few days, basic laboratory testing was unremarkable, except that the blood tacrolimus concentration was 4.56 ng/mL after tacrolimus had been withheld for more than 7 days. He was discharged one day later (Fig. 2).

## Discussion and conclusions

Although a drug–drug interaction with Paxlovid is expected, at the very beginning, we were not sure whether tacrolimus triggered the severe diarrhea. We adjusted the dose of Paxlovid according to the American Society of Transplantation (AST) statement, which suggested reducing the tacrolimus dose to 20% (one-fifth) of the current dose [2]. When we excluded the possibility of intestinal infection and the detection of a high concentration of tacrolimus after stopping it for 7 days, we were confident that the culprit was tacrolimus.

The Paxlovid drug instructions suggest that there should be close monitoring of the concentration of tacrolimus when these drugs are used together. Tang *et al*. summarized nine patients who were prescribed Paxlovid without withholding tacrolimus, which resulted in a surge in tacrolimus concentrations, where the patients later required hospitalization [1]. Coyne *et al*. reported that two renal transplant recipients treated with Paxlovid without adjusting tacrolimus suffered diarrhea [3]. Yanay *et al*. demonstrated that even when reducing the dose of tacrolimus by half when it was concomitant with Paxlovid for only 2 days, an extremely high concentration of tacrolimus could be detected [4]. HHS and diabetic ketoacidosis are life-threatening complications that were triggered by severe diarrhea in this patient. Timely identification, aggressive fluid administration, careful electrolyte replacement ,and intravenous insulin infusion are the key points to manage this emergency situation [5]. Even though this patient recovered gradually without any permanent organ function impairment, he felt unhappy with this adverse event. He thought that what he suffered could have been avoided.

Through our experience (reduced to 25% of the current dose), we do not think a reduction of one-fifth is safe. Thus, we suggest that tacrolimus should be stopped when Paxlovid is applied and that corticosteroids could be a good substitution.

## Data Availability

All data metioned in this paper is available.

## Abbreviations

COVID-19: Moderate coronavirus disease 2019
CYP: Cytochrome P450
SARS-CoV-2: Severe acute respiratory syndrome coronavirus 2
SpO_2_: O_2_ saturation
AST: American Society of Transplantation
Scr: Serum creatinine
BUN: Blood urea nitrogen
